# Hearing aid effectiveness after aural rehabilitation - individual versus group (HEARING) trial: RCT design and baseline characteristics

**DOI:** 10.1186/1472-6963-9-233

**Published:** 2009-12-15

**Authors:** Margaret P Collins, Pamela E Souza, Chuan-Fen Liu, Patrick J Heagerty, Dagmar Amtmann, Bevan Yueh

**Affiliations:** 1 Health Services Research & Development Center of Excellence, VA Puget Sound Health Care System, 1100 Olive Way, Suite 1400, Seattle, WA, 98101, USA; 2 Rehabilitation Care Service, VA Puget Sound Health Care System, 1660 South Columbian Way, Seattle WA, 98108, USA; 3 Northwestern University, Department of Communication Sciences and Disorders, Francis Searle Building 2-265, 2240 Campus Drive Evanston, IL, 60208, USA; 4 Department of Health Services, University of Washington, Box 358280 Health Services, Seattle WA, 98195, USA; 5 Department of Biostatistics, University of Washington, F-667 Health Sciences, Seattle WA, 98195, USA; 6 Department of Rehabilitation Medicine, University of Washington, BB-957 Health Sciences, Seattle WA, 98195, USA; 7 Department of Otolaryngology/Head & Neck Surgery, University of Minnesota, 420 Delaware Street SE, Minneapolis, MN, 55455, USA

## Abstract

**Background:**

Hearing impairment is the most common body system disability in veterans. In 2008, nearly 520,000 veterans had a disability for hearing loss through the Department of Veterans Affairs (VA). Changes in eligibility for hearing aid services, along with the aging population, contributed to a greater than 300% increase in the number of hearing aids dispensed from 1996 to 2006. In 2006, the VA committed to having no wait times for patient visits while providing quality clinically-appropriate care. One approach to achieving this goal is the use of group visits as an alternative to individual visits. We sought to determine: 1) if group hearing aid fitting and follow-up visits were at least as effective as individual visits, and 2) whether group visits lead to cost savings through the six month period after the hearing aid fitting. We describe the rationale, design, and characteristics of the baseline cohort of the first randomized clinical trial to study the impact of group versus individual hearing aid fitting and follow-up visits.

**Methods:**

Participants were recruited from the VA Puget Sound Health Care System Audiology Clinic. Eligible patients had no previous hearing aid use and monaural or binaural air-conduction hearing aids were ordered at the evaluation visit. Participants were randomized to receive the hearing aid fitting and the hearing aid follow-up in an individual or group visit. The primary outcomes were hearing-related function, measured with the first module of the Effectiveness of Aural Rehabilitation (Inner EAR), and hearing aid adherence. We tracked the total cost of planned and unplanned audiology visits over the 6-month interval after the hearing aid fitting.

**Discussion:**

A cohort of 659 participants was randomized to receive group or individual hearing aid fitting and follow-up visits. Baseline demographic and self-reported health status and hearing-related measures were evenly distributed across the treatment arms.

Outcomes after the 6-month follow-up period are needed to determine if group visits were as least as good as those for individual visits and will be reported in subsequent publication.

**Trial Registration:**

NCT00260663

## Background

In 2008, nearly 520,000 veterans had a disability for hearing loss through the Department of Veterans Affairs (VA), making hearing impairment the most common body system disability in veterans [1]. Changes in eligibility for hearing aid services through the Veterans' Health Care Eligibility Reform Act of 1996 (Public Law 104-262) and the Veterans Health Administration (VHA) Directive 96-069 of 1997, along with the aging population, contributed to a greater than 300% increase in the number of hearing aids dispensed from 1996 to 2006 [2]. VHA Directive 2008-070 further expanded eligibility and will likely contribute to additional increases in demand.

Further, in 2006 the VA committed to having no wait times for patient visits while providing quality clinically-appropriate care (VHA Directive 2006-028). Implementation of Systems Redesign (SR) tools were recommended to help accomplish this goal. One of the SR principles aimed at reducing demand is the use of group visits as an alternative to individual visits. For new hearing aid users, the initial orientation and follow-up sessions may be well suited to a group format because they consist of standardized teaching elements and discussion topics relevant to all participants. While group visit are a promising approach, it is important to know that they provide at least equivalent outcomes as individual visits before recommending widespread use of group visits.

Most of the research in audiology group visits has focused on group aural rehabilitation as a supplement to the standard one-on-one visit [3-18], and has suggested that such visits produce equivalent or even better patient outcomes compared to no additional rehabilitation. Some promising non-randomized observational studies suggests that group hearing aid visits can yield patient outcomes (e.g., hearing handicap, hearing-related function, satisfaction, adherence) that are at least as good as or better than the same care provided in an individual format [19-21]. If research using prospective randomized designs shows that group hearing aid visits provide equivalent or better outcomes, then routine use of group visits may be recommended as a means for reducing the strain on resources and waiting times while maintaining quality care. The purpose of this investigation was to conduct such a study by examining the impact of group versus individual hearing aid fitting and group versus individual hearing aid follow-up visits in terms of hearing-related outcomes and treatment costs. In this report, we describe the design of this trial and the baseline characteristics of the randomized cohort.

## Methods

We used a non-inferiority randomized clinical trial with a factorial design to determine: 1) if group visits were at least as effective as individual visits ('non-inferior') for two types of audiology visits (hearing aid fitting and hearing aid follow-up), as measured by hearing-related function and hearing aid adherence six months after hearing aid fitting, and 2) whether group visits led to cost savings through a six month acclimatization period after the hearing aid fitting.

We hypothesized that when compared to individual visits, group hearing aid fitting and follow-up visits would yield: 1) equivalent or improved hearing-related function (primary outcome) and equivalent or improved hearing aid adherence (secondary outcome), and 2) group visits would lead to cost savings from reduced audiology person-hours for initial rehabilitation, and equivalent or improved (lower) rates of unplanned visits in the six-month period after fitting.

**Figure 1:**
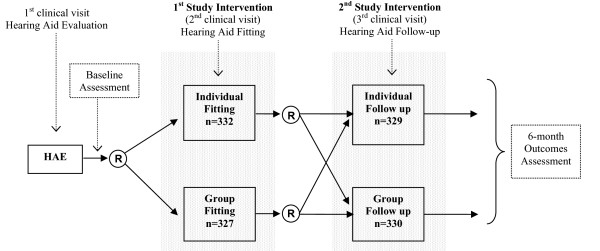
**Trial Design**.

New hearing aid participants were randomly assigned to a group or individual hearing aid fitting, and to a group or individual hearing aid follow-up (Figure 1). Therefore, participants could have received one of four visit combinations (Table 1): individual fit and individual follow-up (I-I); individual fit and group follow-up (I-G); group fit and individual follow-up (G-I); or group fit and group follow-up (G-G). Baseline questionnaires, described below, were completed prior to the hearing aid fitting. Follow-up questionnaires were completed after the fitting and follow-up visits, and six months after the fitting. In addition, participants completed session evaluation and knowledge retention questionnaires immediately after the fitting and follow-up visits. We describe the four types of visits, measures of effectiveness, and other aspects of data collection in detail below. The timing of data collection is summarized in Table 2.

**Table 1:** Hearing aid fit and follow-up visit combinations.

		Fitting Type	
		
		Individual	Group
**Follow-up Type**	**Individual**	I-I	G-I
	
	**Group**	I-G	G-G

**Table 2:** Timing of data collection.

Variable	Source of Data	Baseline	Fit & Follow-up	6-month Follow-up
**Baseline variables and co-variate**				

Co-morbidity	SIC	x		

General health status	SF-12	x		x

Psychiatric conditions	PHQ	x	x	x

Demographics	CPRS	x		

Type, degree of hearing loss	CPRS	x		

**Treatment (rehabilitation) data**				

Session characteristics	Research Assistant		x	

Mediating Mechanisms	Questionnaire		x	

Hearing aid features	CPRS			x

**Costs & Utilization**				

Utilization: unplanned visits	CPRS			x

Costs of planned visits	CPRS, DSS			x

Costs of unplanned visits	CPRS, DSS			x

**Outcomes Assessment**				

Hearing-related function	EAR	x	x	x

Adherence to hearing aids	Two questions		x	x

Hearing handicap	HHIE	x	x	x

Satisfaction with hearing aids	SADL		x	x

Communication Strategies	CPHI	x	x	x

Hearing aid outcome	IOI-HA		x	x

Hearing-related QoL transitional measure	Questionnaire		x	x

CPHI = Communication Profile for the Hearing Impaired, CPRS = Computerized Patient Record System, DSS = Decision Support System, EAR = Effectiveness of Aural Rehabilitation, HHIE = Hearing Handicap Inventory for the Elderly, IOI-HA = International Outcome Inventory - Hearing Aid, PHQ = Patient Health Questionnaire, QoL - quality of life, SADL = Satisfaction with Amplification in Daily Life, SF-12 = SF-12 Health Survey, SIC = Seattle Index of Co-morbidity

### Study Population

Study participants were recruited from the Audiology Clinics at the Seattle and American Lake Divisions of the Puget Sound Health Care System from February 2006 through October 2007. Participants were eligible to enroll if they met the following criteria:

• No previous hearing aid use

• Monaural or binaural air-conduction hearing aids were fit as a result of findings at the hearing aid evaluation visit. Under the Veterans' Health Care Eligibility Reform Act of 1996 (Public Law 104-262) and the Veterans Health Administration Directive 96-069 of 1997, participants were eligible for VA hearing aids if they had a 10-100% disability rating for any medical condition, any disability rating for a hearing related condition, receiving aid and attendance or housebound benefits from the VA, former Prisoner of War, awarded a Purple Heart, receipt of benefits under Title 38, hearing loss resulting from another medical condition for which the veteran was receiving on-going VA care or that resulted from treatment of that condition, or hearing loss severe enough to interfere with the ability to participate actively in their own medical care

We excluded participants who met the following criteria:

• previous hearing aid use

• unable to participate in group instruction because of cognitive or physical restrictions

• unable to give informed consent

• unable to complete self-administered questionnaires written in simple English

• unwilling or unable to return for follow-up visit or complete follow-up questionnaires

### Recruitment, Randomization, and Baseline Assessment

#### Recruitment

All participants being seen for a hearing aid evaluation were given a study flyer when they checked in for their hearing evaluation visit. The flyer briefly described the study and eligibility criteria. At the conclusion of the evaluation, participants who met the inclusion criteria were offered enrollment by the audiologist who conducted the evaluation. Participants who expressed interest were directed to an on-site research assistant (RA). The RA explained the study in detail in a quiet, private room and conducted the process of informed consent. Participants were offered $50 ($10 for each set of questionnaires completed, and an additional $10 for completing all 4 questionnaires at the end of the 6-month period) as remuneration for the time and effort of participating. Remuneration was made with one payment six months after the hearing aid fitting.

#### Randomization

Participants were randomized to group or individual hearing aid fitting and to group or individual hearing aid follow-up. Randomization assignments were prepared ahead of time in variable blocks of four or eight participants, and then placed in sealed, opaque envelopes. Thus, RAs did not know which treatment participants would receive. After randomization, participants were scheduled for the hearing aid fitting and follow-up appointments according to their randomization assignments. Participants returned for the fitting three-to-four weeks after the evaluation. The follow-up appointment was scheduled for three-to-five weeks after the fitting.

#### Baseline Assessment

After enrollment, baseline co-variates were collected and baseline assessment surveys were administered. Participants who did not have time to complete the surveys that day completed them at home and returned them in a postage-paid envelope prior to the hearing aid fitting.

##### Hearing-related outcomes

• The Effectiveness of Auditory Rehabilitation (EAR) [22] measures hearing-related function with two separate modules. We used the first module at baseline. This 10-item module (the Inner EAR) covers intrinsic hearing issues such as hearing in quiet and hearing in noise. It is scored from 0 to 100, with *higher *scores representing *better *function. The Inner EAR has an additional global question scored on a 0-10 scale. The second module (Outer EAR) covers hearing-aid related issues such as comfort, appearance, and convenience, is relevant only after hearing aid fitting and therefore was not administered at the baseline visit. Both modules have excellent psychometric reliability, validity, and responsiveness.

• Hearing-related handicap was assessed with the Hearing Handicap Inventory for the Elderly (HHIE) [23-27] questionnaire. Scores reflect social and emotional hearing handicap with social and emotional hearing-handicap domain scores. The scale is scored from 0 to 100, with *higher *scores representing *more *handicap.

• The Communication Strategies scales of the Communication Profile for the Hearing Impaired (CPHI) [28-31] was used to assess participants' behavior that may facilitate or hinder effective communication. Three subscales are used to assess use of maladaptive behaviors, verbal strategies, and nonverbal strategies during communication. These scales have well-know psychometric properties and consist of 25 questions scored on a 5-point scale. *Higher *scores indicate *better *use of communication strategies.

##### Baseline co-variates

• Co-morbidity is a potentially confounding variable because those with greater disease burden tend to have worse health-related quality of life. We used the Seattle Index of Co-morbidity (SIC) [32] which is a validated outpatient co-morbidity score developed at VA Puget Sound Health Care System using self-identified chronic medical conditions. *Higher *scores indicate *more *co-morbitity.

• Health status, like co-morbidity, may be a confounding variable because of its independent effect on quality of life. We assessed general health status with the SF-12 [33]. This scale yields a physical component score (PCS-12) and mental component score (MCS-12), reflecting general physical and mental health status. *Higher *scores indicate *poorer *health.

• The Patient Health Questionnaire (PHQ) [34] was used to assess several mental health constructs. Depression and depressive symptom severity were measured with the 9-item depression module which are explicitly tied to Diagnostic and Statistical Manual of Mental Disorders-IV (DSM-IV) diagnostic criteria for depression [35]. In addition, we used the PHQ to assess anxiety and alcohol abuse, both of which may influence receptivity to group involvement or follow-up adherence. The PHQ is a widely-used, well-validated questionnaire with high reliability and construct validity

• Demographic data. The VA Computerized Patient Record System (CPRS) was used to obtain age, service-connection rating, and gender.

• Audiometric data obtained at the hearing aid evaluation, such as type and degree of hearing loss were also abstracted from CPRS.

### Group and Individual Interventions

#### Hearing aid fitting

The first randomized intervention was the orientation portion of the hearing aid fitting appointment. All participants, regardless of randomization, received their aids during an individual 45-minute session at which time the aids were programmed for the participant's specific requirements. Hearing aid amplification characteristics were adjusted to match NAL-NL1 [36] hearing aid prescription targets and verified with real-ear measures. The use of specific hearing aid features such as a volume control, memory button, directional microphone, and remote control were explained during this programming session. After the 45-minute individual session, participants received either group or individual orientation as described below. Participants' significant others were invited to attend all appointments.

#### Group fitting

For the group fitting, up to three participants received individual programming during one 45-minute period. They then waited while a second set of up to three participants received programming during a second 45-minute session. Then, these six participants and their guests convened for a 60-minute group orientation in a quiet conference room in or near the audiology clinic. The orientation was conducted by a hearing aid technician or audiologist. Teaching topics included: how a hearing aid works; use, care, and maintenance; acoustic feedback; battery use, replacement, and ordering; telephone use; warranty and repairs; listening and communication strategies; and realistic expectations. Functions of special features covered during the individual portion of the visit were reviewed. The format was standardized and facilitated by a PowerPoint presentation combined with hands-on practice. Participants also received a handout of the PowerPoint presentation. An example of the timing of a typical group hearing aid fitting is shown in Table 3. In this scenario, 4.5 hours of audiologist time and 1 hour of audiologist or technician time was used to see up to six participants.

**Table 3:** Group hearing aid fitting clinic schedule.

HAF appointment	Duration	Audiologist A	Audiologist B	Audiologist C	Technician or Audiologist
Individual Programming	45 minutes	Patient #1	Patient #2	Patient #3	

Individual Programming	45 minutes	Patient #4	Patient #5	Patient #6	

Group Orientation	60 minutes	--	--	--	Patients #1-6

#### Individual fitting

This orientation was provided to the participant by the same audiologist who performed the programming, immediately following and in the same room as the programming. This session typically took 30 minutes. The same topics were covered as in the group orientation, without the aid of the PowerPoint presentation; however, a printout of the PowerPoint presentation was provided to these participants. Therefore, participants in both the individual and group randomization arms received the same information. Nonetheless, because of the individualized nature of the session, some information may have varied from participant to participant, as well as from audiologist to audiologist, depending on the participant's specific needs and requests. An example of the timing of a typical individual hearing aid fitting is shown in Table 4. In this scenario, 7.5 hours of audiologist time was used to see up to six participants.

**Table 4:** Individual hearing aid fitting clinic schedule.

HAF appointment	Duration	Audiologist A	Audiologist B	Audiologist C
Individual Programming Individual Orientation	45 minutes 30 minutes	Patient #1	Patient #2	Patient #3

Individual Programming Individual Orientation	45 minutes 30 minutes	Patient #4	Patient #5	Patient #6

#### Hearing Aid Follow-up Visit

The second randomized intervention was the follow-up visit that was scheduled for three to five weeks after the fitting, depending on appointment availability.

#### Group follow-up

This visit was scheduled with up to five participants during a 75-minute sessions, 45 minutes of which reviewed information covered at the hearing aid orientation, and covered the effects of acquired hearing loss, coping with hearing loss, pinpointing the source of communication problems, realistic expectations, management of difficult communication situations, guidelines for hard of hearing, and guidelines for significant others. These visits were conducted by one of the staff audiologists in the same quiet conference room in or near the audiology clinic as the group orientation visits. This session was facilitated by a PowerPoint presentation, and participants were provided with a handout of the presentation. Individual adjustments, if needed, were conducted during the 30 minutes after the group portion. In this scenario, 75 minutes of audiologist time was used to see up to five participants (Table 5). For some weeks, the five-person group was not filled resulting in a smaller group size.

**Table 5:** Group hearing aid follow-up clinic schedule.

Follow-up appointment	Duration	Audiologist A	Audiologist B	Audiologist C
Group	75 minutes	Patients #1-5	--	--

#### Individual follow-up

This visit consisted of an individual 30-minute visit with an audiologist to discuss problems and to make hearing aid adjustments when appropriate. Three person-hours of audiology time were required to see 6 participants (Table 6). Effective hearing aid use and communication strategies were discussed as necessary depending on the individual's specific concerns. As with the individual fitting visits, a hard copy of the same PowerPoint presentation was used to guide the discussion and a copy was provided to participants.

**Table 6:** Schedule for an individual hearing aid follow-up clinic.

Follow-up appointment	Duration	Audiologist A	Audiologist B	Audiologist C
Individual	30 minute	Patient #1	Patient #2	Patient #3

Individual	30 minute	Patient #4	Patient #5	Patient #6

### Follow-up Assessments

#### Post-hearing aid fitting and post-follow-up

Follow-up surveys were mailed to participants 10 days after the hearing aid fitting visit and two weeks after the follow-up visit. Questionnaires were returned by postage-paid mail. The scales were checked for completeness by the RA before the fitting and follow-up visit; participants who forgot to return their packets were given a second opportunity to complete the scales while waiting for their visits. In addition to the Inner EAR, HHIE, and CPHI described above, the following surveys were mailed:

• The Outer EAR, introduced above, is a 10-item self-rated scale [22]. Like the Inner EAR, it is also scored from 0 to 100, with *higher *scores representing *better *function. It also has two global questions. This module has also been demonstrated to have excellent reliability, validity, and responsiveness to clinical change.

• The Adherence to Hearing Rehabilitation (AdHeRe) questionnaire measures hearing aid adherence with both dichotomous ("Do you use your hearing aids?") and continuous responses ("How many hours a day do you use your hearing aid?"). Both types of responses have good construct validity with hearing related function and hearing handicap [37,38].

• The Satisfaction with Amplification in Daily Life (SADL) [39,40] is a 15-item self-report scale that measures satisfaction with hearing aids. The SADL provides a global score reflecting overall satisfaction and four subscales addressing positive effects, service and cost, negative features, and personal image [39]. Item #14 regarding the cost of hearing aids was eliminated because veterans do not purchase their aids. Scores range from 1 to 7, with *7 *representing *maximum *satisfaction. Construct and internal validity of the SADL are strong [40].

• The International Outcome Inventory for Hearing Aids (IOI-HA[41]) is a general self-assessment tool that was developed for use as an addendum to research protocols. The hearing research community has proposed that this scale be applied to facilitate comparison of hearing aid outcomes across diverse research studies. It is a brief 8-item survey, with reasonably strong internal consistency and psychometric properties. Items are scored on a 5-point Likert scale with *higher *scores reflecting *better *outcomes.

• Transitional measure of hearing-related quality of life (QoL). Transitional outcome measures have been shown to be more sensitive to change than serial measures taken over several points in time [42]. This single item instrument asked participants to indicate how much their hearing-related quality of life changed in the past year. It uses two 7-point Likert scales to distinguish change and is adapted from the work of Gordon Guyatt and colleagues at McMaster University ("Global Rating of Change") [43,44].

#### Treatment (rehabilitation) data

Immediately after the fitting visit and after the follow-up visit, an RA distributed a session evaluation and knowledge retention questionnaire. The session evaluation questionnaire consisted of 11 questions pertinent to all participants regarding various mechanisms that might mediate the effectiveness of rehabilitation such as presence of family members, amount of attention and time spent with providers, session pace and information repetition, opportunities for questions, and discomfort in social situations. Four additional questions that were directed only to participants who had a group visit asked about privacy concerns, effectiveness of the PowerPoint slides, group interactions and shared insights. The knowledge retention questionnaire asked 12 questions about standard information that should have been learned in the session such as what color indicates the right and left hearing aids. The RA also collected data about the session characteristics such as how many participants and or spouses were present, length of session, wait time between programming and orientation, etc. Six months after the fitting, features of the hearing aid(s) (e.g., style, ear, presence of volume control, presence of multi-memory, etc) was obtained from CPRS chart review.

#### Six-month follow-up

Six months after the fitting visit, we mailed the final set of outcomes questionnaires to participants. These were returned by postage-paid mail. The packet consisted of the SF-12, PHQ, EAR (Inner and Outer), HHIE, CPHI, AdHeRe, SADL, IOI-HA, and transitional QoL measure. If items were left blank, an RA attempted to obtain the information via telephone.

### Utilization and Cost Outcomes

We chose the 6-month interval after the hearing aid fitting to track utilization and cost variables because pilot data indicated that 75% of all unplanned visits occurred in this timeframe. Total costs will be the sum of costs of planned (fitting and follow-up) and unplanned (e.g., hearing aid repairs) audiology visits. We estimated the cost of fitting and follow-up visits based on provider time and pay grade. For unplanned visits, we estimated the cost of the visit using the cost for each CPT code associated with the visit from the VA cost accounting system. All cost estimates included fringe benefits and indirect and overhead costs.

### Data and Study Management

The study and all study modifications were approved by the Institutional Review Board of the University of Washington and the Research and Development Committee at VA Puget Sound Health Care System. RAs received thorough training in the process of informed consent, and their first enrollment interview received full edit reviews. Ten percent of all participant files were randomly audited for errors by the project manager. Data were entered into a Microsoft Access database using double-data entry verification.

### Analysis

The cohort description and baseline characteristics are described in this report using straightforward descriptive statistics. Future study results will use an intent-to-treat analysis. The primary purpose of this trial was to investigate the relative effectiveness of group and individual appointments in audiology. We used a factorial design with concurrent testing of group fittings and group follow-ups to facilitate insight into the value of a group format for both visits with one trial. The primary outcome of effectiveness was hearing-related function (Inner EAR) 6 months after the hearing aid fitting.

For our primary analysis, we will evaluate the overall effectiveness of group versus individual hearing aid fitting and group versus individual hearing aid follow-up for the Inner EAR using multivariate mixed model regression to adjust for factors related to hearing-related function and account for potential clustering among participants of a given group visit. Because our trial was a non-inferiority study, we will test the one-sided hypothesis that the effect of group visits does not lead to a detrimental effect beyond the clinically significant range. Secondary analyses will focus on secondary outcomes, and also on subgroup analyses for the primary outcome variable. Secondary outcomes will include hearing aid adherence, hearing handicap, communication strategies, and satisfaction with amplification. Subgroup analyses will focus on principal covariates such as use of monaural versus bilateral aids, sensorineural versus mixed hearing loss, degree of hearing loss, and the presence of specific hearing aid features such as directional microphones and telephone coils. The study was adequately powered to detect large differential treatment effects, but analyses of smaller effects will be exploratory rather than confirmatory.

Economic evaluation will estimate potential cost savings from the VA's perspective for group visits compared to individual visits. We will examine the effect of the group visit intervention on the number of unplanned visits and cost of unplanned visits. Although this study was not powered to definitively determine cost-effectiveness, exploratory calculations of the unit cost to obtain a successfully treated patient (an Inner EAR improvement of more than the minimally clinically important difference of 6.0 points) will be made. We will also conduct analyses with a secondary measure of treatment success: adherence to hearing aid use (the cost to obtain an adherent patient, and the cost to obtain an additional hour of adherence per day).

### Sample Size

We estimated our sample size based on a minimum clinically significant difference in Inner EAR scores of 6.0 points on a 100 point scale with standard deviation of 23.5, and a conservative loss to follow-up of 20%. We controlled for possible provider effects, site, and treatment, and accounted for most sources that induce clustering, and thus anticipate weak (<.10) within-group correlation. Enrollment of 660 participants (330/arm) would yield 90% power (and an alpha error of 5%) to reject the one-sided null hypothesis. An important consideration was to ensure that we had adequate power to detect changes in secondary outcomes, such as decreases in dichotomous hearing aid adherence. Using a sample size of 264 analyzable participants in each arm and our baseline adherence rate of 95%, we will have 92% power to ensure that hearing aid adherence rates do not worsen more than 10% (relative percentage).

Our study design was a factorial trial evaluating two interventions. This has the benefit of research economy and the ability to test for non-additive effects for the two interventions. For our research questions we chose not to make a multiple comparison adjustment since we are prepared to make separate conclusions for each of the two interventions. A multiple comparison correction is appropriate whenever a single conclusion is derived from the evaluation of multiple tests. We will make separate conclusions regarding group versus individual hearing fitting and regarding group versus individual follow-up.

### Results: Baseline

#### Enrollment

A cohort of 659 participants was enrolled from February 2006 through October 2007. Eleven participants requested to be dropped from the study, one died prior to the fitting, and three others were later determined to be ineligible, leaving 644 participants. Of these, 323 and 321 participants were randomized to individual and group fittings, respectively, and 324 and 320 participants randomized to individual and group follow up, respectively. This resulted in four different visit combinations (Table 1): 164 were randomized to Individual fitting visits and Individual follow-up visits ('I-I category'), 160 to Group fitting and Individual follow-up ('G-I category'), 159 to Individual fitting and Group follow-up ('I-G category'), and 161 to Group fitting and Group follow-up ('G-G category').

#### Baseline Characteristics

**Figure 2:**
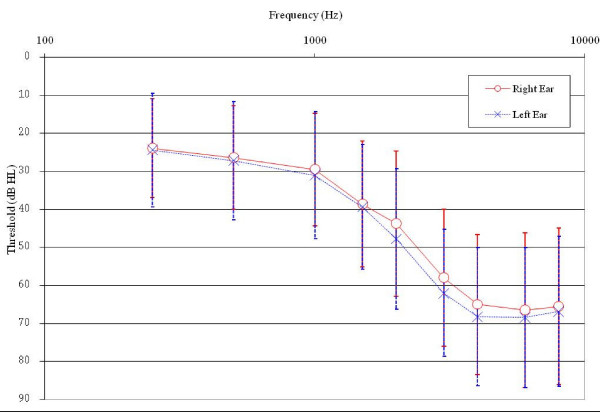
**Average hearing loss for participants at baseline**. Vertical bars represent +/- one standard deviation.

All baseline demographic and hearing characteristics were evenly distributed across the four visit combinations (Table 7). Most participants were men (98.5%) with a mean age of 65.5 years. The average hearing loss (Figure 2) was a mild sloping to moderately severe hearing loss from 250 Hz through 8000 Hz, with the left ear high-frequency average (average of 1000, 2000, 3000, and 4000 Hz) 3.2 dB worse than the right ear average. This left-ear asymmetry is a common hearing loss pattern in veterans resulting from more noise exposure to the left ear with right-handed fire arm use. Monosyllabic word recognition (Maryland CNC words) was good for both ears with an average of 88.6% correct in the right ear and 87.0% correct in the left ear. Almost all losses were bilateral (98.0%) and sensorineural with only 5.1% and 4.1% of losses on the right and left, respectively, being conductive, mixed, or normal.

**Table 7:** Baseline demographic and hearing characteristics for participants in the four appointment groups.

Characteristic	I-I (n = 164)	G-I (n = 160)	I-G (n = 159)	G-G (n = 161)	*p*-value
Gender (% male)	98.2	100.0	98.1	97.5	0.30
Age (mean years ± SD)	65.2 ± 10.5	65.4 ± 11.6	66.2 ± 10.6	65.3 ± 11.5	0.84
Right-ear HF PTA (dB HL ± SD)	48.8 ± 14.6	50.0 ± 15.8	48.4 ± 14.5	48.9 ± 14.2	0.80
Left-ear HF PTA (dB HL ± SD)	53.0 ± 15.1	51.8 ± 14.4	53.1 ± 15.0	50.9 ± 13.4	0.48
Right-ear word recognition (mean % correct ± SD)	88.7 ± 13.4	87.3 ± 13.8	90.2 ± 11.0	88.1 ± 14.1	0.80
Left-ear word recognition (mean % correct ± SD)	87.5 ± 14.5	86.6 ± 12.3	86.8 ± 13.6	87.1 ± 13.9	0.48
Unilateral loss (%)	2.4	1.3	2.5	1.9	0.84
Right-ear SNHL (%)	95.7	96.3	93.0	94.4	0.74
Left-ear SNHL (%)	96.9	96.9	94.9	95.0	0.63

HF PTA = high-frequency pure-tone average (average of thresholds at 1000, 2000, 3000, and 4000 Hz), SD = standard deviation, SNHL = sensorineural hearing loss

All health status characteristics were also evenly distributed across the four visit combinations (Table 8). The mean SF-12 PCS and MCS scores of 34.6 and 44.2, respectively, were in the bottom 25^th ^percentile of normative scores for men in the general U.S. population age 65-74 [33]. The mean SIC score of 6.2 indicated that our cohort was representative of the veteran population [32]. Scores on the PHQ showed that 13.1%, 5.7%, 12.3%, 10.6%, and 12.1% of participants were experiencing major depression, other depression, panic, anxiety, and excessive alcohol use, respectively.

**Table 8:** Baseline self-assessed health status characteristics in the four different treatment combinations.

Characteristic	I-I	G-I	I-G	G-G	*p*-value
SF-12					
PCS (mean score ± SD)	34.2 ± 8.1 (160)	35.0 ± 8.3 (153)	34.2 ± 7.9 (154)	35.1 ± 8.3 (151)	0.64
MCS(mean score ± SD)	44.6 ± 9.2 (160)	44.3 ± 8.8 (153)	44.2 ± 10.7 (154)	43.7 ± 9.5 (151)	0.86
SIC (mean score ± SD)	6.2 ± 3.4 (164)	6.3 ± 3.3 (160)	6.4 ± 3.5 (159)	6.1 ± 3.3 (161)	0.90
PHQ					
% with Major Depression	11.2 (161)	13.6 (155)	15.1 (159)	12.7 (157)	0.77
% with Other Depression	5.0 (161)	6.5 (155)	4.4 (159)	7.0 (157)	0.72
% with Panic	11.8 (161)	9.0 (155)	12.6 (159)	15.9 (157)	0.32
% with Anxiety	8.7 (161)	10.3 (155)	12.6 (159)	10.8 (157)	0.73
% with Excessive Alcohol	13.7 (161)	9.7 (155)	17.1 (158)	7.7 (156)	0.05

Number of participants shown in parentheses.

MCS = mental component score, PCS = physical component score, PHQ = Patient Health Questionnaire, SD = standard deviation, SF-12 = SF-12 Health Survey, SIC = Seattle Index of Co-morbidity,

Self-reported hearing-related function scores are shown in Table 9 and were not statistically different across the four visit combinations. The average Inner EAR score was 27.3. This score was consistent with the average score of 25.7 found for 112 first-time hearing aid users at the VA Puget Sound Health Care System in the SAI-WHAT hearing screening trial (.)[45]. Hearing handicap was assessed with the HHIE. The overall average total score of 48.1 indicated a moderate degree of perceived handicap that is in line with unaided findings for first-time hearing aid users. Research by Weinstein, Spitzer, and Ventry [46] showed that 27 mostly VA Audiology participants with a mean age of 80 years had an average total score of 57.4 (SD = 30.9) and social and emotional scores of 25.5 and 29.1, respectively. Similar to their finding, we also found that overall handicap was fairly equally distributed between the social and emotional handicap domains. For the CPHI, our cohort showed some limited use of maladaptive behaviors such as avoiding difficult listening situations or pretending they heard, and somewhat poorer verbal and nonverbal strategies such as asking watching the speaker's face or asking them to repeat. These findings are similar to those from large samples of active-duty military personnel with mostly bilateral, noise-induced, high-frequency sensorineural hearing loss who participated in the CPHI development [28,31], For example, Demorest and Erdman [31] reported scores of 3.95 (SD = 0.67), 2.97 (SD = 0.76), and 3.69 (SD = 0.74) for the maladaptive behaviors, verbal strategies, and nonverbal strategies subscales, respectively, from a cohort of 1226 active-duty military personnel who participated in an Aural Rehabilitation program at the Walter Reed Army Medical Center from 1985-1987. In general, our self-reported hearing-related results are consistent with expected values from an unaided hearing-impaired population.

**Table 9:** Means ± standard deviations for baseline self-assessed hearing-related characteristics.

Characteristic	I-I	G-I	I-G	G-G	*p*-value
**Inner EAR**	25.1 ± 13.1 (160)	28.6 ± 14.7 (155)	28.9 ± 14.4 (158)	26.9 ± 13.6 (157)	0.06
**HHIE**					
Social	24.5 ± 10.6 (160)	23.5 ± 11.4 (152)	23.0 ± 11.8 (156)	24.2 ± 11.0 (154)	0.63
Emotional	25.2 ± 12.5 (161)	23.9 ± 13.4 (151)	22.7 ± 14.1 (156)	25.3 ± 13.3 (154)	0.27
Total	49.7 ± 22.4 (160)	47.3 ± 23.6 (148)	45.8 ± 24.9 (155)	49.8 ± 23.0 (152)	0.36
**CPHI**					
Maladaptive Behaviors	4.0 ± 0.7 (161)	4.0 ± 0.7 (155)	4.1 ± 0.8 (158)	4.0 ± 0.8 (156)	0.72
Verbal Strategies	2.7 ± 0.8 (161)	2.8 ± 0.9 (155)	2.6 ± 0.9 (158)	2.9 ± 0.9 (156)	0.05
Non-Verbal Strategies	3.3 ± 0.9 (161)	3.4 ± 0.9 (155)	3.3 ± 1.0 (158)	3.5 ± 0.9 (156)	0.28
Overall Score	3.4 ± 0.5 (161)	3.4 ± 0.5 (155)	3.4 ± 0.5 (158)	3.5 ± 0.5 (156)	0.15

Number of participants shown in parentheses.

CPHI = Communication Profile for the Hearing Impaired, EAR = Effectiveness of Aural Rehabilitation, HHIE = Hearing Handicap Inventory for the Elderly

## Discussion

We conducted a non-inferiority randomized clinical trial with a factorial design to determine if group hearing aid fitting and hearing aid follow-up visits were at least as effective as individual visits ('non-inferior'). Our study was designed to detect differences in hearing-related function and cost throughout the six-month acclimatization period following the hearing aid fitting for each of these visit types. We randomized 659 eligible participants from February 2006 through September 2007 at the Seattle and American Lake Divisions of VA Puget Sound Health Care System to receive group or individual hearing aid fitting and group or individual hearing aid follow-up. At baseline, all measures for 644 active participants were evenly distributed across the visit combinations and representative of an older male hearing-impaired veteran population seeking their first pair of hearing aids.

Data from randomized trials represent the highest level of evidence about the effectiveness of interventions [47]. To our knowledge, the HEARING trial is the first randomized trial to study the effectiveness of group versus individual hearing aid fitting and follow-up visits. The study was powered to detect clinically significant differences in hearing-related function and cost between group and individual visits. A number of secondary outcomes also will be considered including hearing aid adherence, hearing handicap, communication strategies, and satisfaction with amplification.

In addition, factors that might mediate the effectiveness of group sessions will also be considered [17,19,48-60]. Potentially positive mediators of group sessions include more overall time with a provider since group sessions are typically extended as a result of time savings from reduced individual appointments. Meeting in group format also reduces the stigma of disease when participants see others with similar problems. Potentially negative mediators include less one-on-one time with the provider. Some participants may sense a loss of privacy or be uncomfortable with the social setting of group visits. There will also be mediators with as yet uncertain impact such as the size of the group. The presence of guests could either improve (pleasant guests) or worsen (annoying guests) the learning environment. Participants may feel they have more attention from the provider because of longer visits, or less attention because of less one-on-one contact. Satisfaction with the session and provider may similarly be affected positively or adversely. Group sessions may influence self-efficacy and expectations, but it is not clear whether this effect will be positive or negative. Group interactions may also either improve or worsen the overall learning environment. Knowledge could be improved if participants benefit from hearing others' questions, or worsened if confusion is introduced when they hear about hearing aid features they do not have.

Our study was designed to determine whether positive or negative effects will predominate on average. If positive effects predominate, or uncertain effects turn out to be positive, we would expect to see better treatment effectiveness (improved hearing-related function, better hearing aid adherence). Conversely, if negative effects predominate, or the bulk of the uncertain effects are negative, we will see worse treatment effectiveness. By gathering data about the potential mediators, we can examine whether these effects exist and how these effects may affect outcomes, giving us preliminary insight into the mechanisms by which group visits may or may not be effective.

This is the first randomized trial to consider the effects on cost from providing group hearing aid visits. Costs from implementation of group visits can be incurred immediately and on a longer-term basis. Immediate cost savings can be realized with the reduced audiology person-hours required during group fitting and follow-up visits. However, the impact on longer-term costs over the rehabilitation period is less clear. If group treatment is effective, there may be more learning and information retention that leads to long-term savings from fewer subsequent unplanned visits and hearing aid repairs. On the other hand, if group sessions are less effective and participants do not learn how to use their aids optimally, the result may be more unplanned visits and more hearing aid repairs. It is also entirely possible that mixed effects will be observed: for example, improved effectiveness at greater cost. The desirable finding that effectiveness is either improved or equivalent (non-inferior) while saving costs was the hypothesis for this study.

Based on prior studies, we expect that group hearing aid visits will yield equivalent or better outcomes compared to individual visits, but with lower costs associated with group visits as a result of fewer providers required to provide the care. Similar findings have been reported for group visits in chronic disease management. With the use of group visits, a number of large managed health care organizations have found lower costs [57,61,62] and reduced utilization [57,62-64], and improved participant satisfaction [57,64-66], self efficacy [62,64], health status, [55,56,61,64,66-69], compliance [54], quality of life [62] and physician satisfaction [57].

Prior to the HEARING trial, no randomized trials have compared group versus individual hearing aid visits; however, three non-randomized studies suggest that group visits may yield better participant outcomes than individual visits [19-21]. Collins, Souza, O'Neill, and Yueh [21] conducted a retrospective (non-randomized) medical chart review of veterans seeking hearing aids at the VA Puget Sound Health Care System from September 2004 to March 2005. This was a time period when the clinic was using both individual and group visits and participants were sequentially assigned to a group visit until that clinic was full, and then to an individual visit. Hearing-related outcome questionnaires were compared between participants seen for individual versus group fitting and follow-up visits. For 74 participants who had completed at least one self-report outcome survey after the follow-up visit, participants who received both fitting and follow-up in group format reported similar hearing handicap, and statistically and clinically better hearing-related function, satisfaction, and adherence than participants who received only individual visits. Brickley, Cleaver and Bailey [19] found no difference in hours of hearing aid use or hearing aid satisfaction in a retrospective study comparing 49 participants who received an individual visit to 49 participants who received a group visit. However, the group sessions cost less to conduct, and participants returned for fewer unplanned visits and reported better performance. Taylor [20] found that participants who received an 8-10 hour aural rehabilitation program in a group reported less hearing handicap and more satisfaction with their audiologist than compared to participants who receive the program on an individual basis.

A number of factors may have influenced how participants in this study responded to group visits. In clinical settings, patients often wait many years from first noticing a hearing loss until they first visit an audiologist for evaluation. However, veterans receive hearing aids free of charge so their threshold for seeking their first pair of hearing aids may be lower that that found in private clinics, possibly yielding new VA hearing aid users with less hearing loss than new private sector users. Degree of impairment may differentially affect receptiveness to group intervention. In addition, receiving hearing aids free of charge may change an individual's motivation and willingness to participate in a group format. For example, if the participant preferred an individual visit, they might be more likely to accept a group visit instead if they received the aids at no charge. In addition, only veterans who were eligible to receive VA hearing aids were enrolled in this study. This means that VA hearing aid participants have sustained some type of service-related disability, and/or have a substantial enough hearing loss to prevent them from participating in their medical care. Because of their military service experiences and/or the severity of their hearing loss, these veterans may respond differently to a group setting. Given these factors, our results may not be generalizable to a non-veteran population.

In addition, it was not possible to blind participants or providers to treatment allocation, possibly biasing participants' perception of their treatment depending on preconceived ideas about group visits. The extent to which group hearing aid visits yield positive outcomes will depend on how cost and effectiveness interact. These data will be reported in a subsequent publication.

## Abbreviations

AdHeRe: Adherence to Hearing Rehabilitation; CPHI: Communication Profile for the Hearing Impaired; CPRS: VA Computerized Patient Record System; DSM-IV: Diagnostic and Statistical Manual of Mental Disorders; EAR: Effectiveness of Aural Rehabilitation; G-G: group fitting and group follow-up; G-I: group fitting and individual follow-up; HEARING: Hearing Aid Effectiveness after Aural Rehabilitation-Individual versus Group; HHIE: Hearing Handicap Inventory for the Elderly; I-G: individual fitting and group follow-up; I-I: individual fitting and individual follow-up; IOI-HA: International Outcome Inventory for Hearing Aids; MCS: Mental Component Score; NAL-NL1: National Acoustic Laboratories-non-linear; PCS: Physical Component Score; PHQ: Patient Health Questionnaire; QoL: Quality of Life; RA: Research Assistant; SADL: Satisfaction with Amplification in Daily Life; SAI-WHAT: Screening for Auditory Impairment: Which Hearing Assessment Test; SIC: Seattle Index of Co-morbidity; SR: Systems Redesign; VA: Veteran's Affairs; VHA: Veterans Health Administration.

## Competing interests

The authors declare that they have no competing interests.

## Authors' contributions

MC participated in the study design and coordination, statistical analysis, and drafted the manuscript. PS participated in the study design and manuscript preparation. CL participated in the study design, cost analysis, and manuscript preparation. PH and DA participated in the study design. BY participated in the study design, coordination, and statistical analysis. All authors read and approved the final manuscript.

## Pre-publication history

The pre-publication history for this paper can be accessed here:

http://www.biomedcentral.com/1472-6963/9/233/prepub
